# An Intelligent Method for Predicting Pacific Oyster (*Crassostrea gigas*) Freshness Using Deep Learning Fused with Malondialdehyde and Total Sulfhydryl Groups Information

**DOI:** 10.3390/foods12193616

**Published:** 2023-09-28

**Authors:** Tao Lu, Fanqianhui Yu, Baokun Han, Jingying Guo, Kunhua Liu, Shuai He

**Affiliations:** 1 School of Mechanical and Automotive Engineering, Qingdao University of Technology, Qingdao 266520, China; lutao@qut.edu.cn (T.L.);; 2 Key Laboratory of Industrial Fluid Energy Conservation and Pollution Control, Qingdao University of Technology, Ministry of Education, Qingdao 266520, China; 3 Haide College, Ocean University of China, Qingdao 266100, China; 4 College of Food Science and Engineering, Ocean University of China, Qingdao 266003, China; 5 Department of Computer Science and Technology, Ocean University of China, Qingdao 266100, China; 6 College of Mechanical and Electronic Engineering, Shandong University of Science and Technology, Qingdao 266000, China; 7 College of the Arts and Society, Coventry University, Coventry CV1 5FB, UK

**Keywords:** oyster, convolutional neural network, freshness prediction, feature visualization, strongest activations

## Abstract

To achieve a non-destructive and rapid detection of oyster freshness, an intelligent method using deep learning fused with malondialdehyde (MDA) and total sulfhydryl groups (SH) information was proposed. In this study, an “MDA-SH-storage days” polynomial fitting model and oyster meat image dataset were first built. AleNet-MDA and AlxNet-SH classification models were then constructed to automatically identify and classify four levels of oyster meat images with overall accuracies of 92.72% and 94.06%, respectively. Next, the outputs of the two models were used as the inputs to “MDA-SH-storage days” model, which ultimately succeeded in predicting the corresponding MDA content, SH content and storage day for an oyster image within 0.03 ms. Furthermore, the interpretability of the two models for oyster meat image were also investigated by feature visualization and strongest activations techniques. Thus, this study brings new thoughts on oyster freshness prediction from the perspective of computer vision and artificial intelligence.

## 1. Introduction

As a popular edible shellfish in the world, oysters are rich in protein (39.1–53.1% on a dry basis) and have the reputation of “milk of the sea” [1,2]. Unfortunately, oysters are a short shelf-life product because they are easily perishable during storage and transportation, especially for the growing market of raw oysters on the half-shell or oyster meat, where rapid undesirable changes in external and internal properties significantly reduce their freshness and safety for consumption [3,4,5]. The commonly used methods to characterize the storage quality of oysters can be classified as sensory, chemical and microbial population evaluations, specifically including assessment of oyster appearance and texture properties, measurement of gross composition and pH changes, analysis of lipid oxidation and protein degradation and counting of bacterial colonies [5,6,7]. In general, these quality evaluation indices are primarily determined by experiment-based laboratory methods that are highly dependent on experimenters’ repetitive sample preparation and measurements [8]. For instance, measuring the total volatile basic nitrogen (TVB-N) content in oysters is destructive, time-consuming and environmentally unfriendly to some extent, as well as difficult to be extended on an industrial scale [9].

With the advancement of technology, several non-destructive and rapid instrumental methods have been developed to evaluate oyster freshness, such as near-infrared reflectance (NIR) spectroscopy and hyperspectral imaging (HSI) [7,10,11]. Notably, Chen et al. [10] combined analytical instrument with computer technique, i.e., used back-propagation artificial neural network (BP-ANN) to predict TVB-N values based on hyperspectral images of oysters. Interdisciplinary research applying artificial intelligence (AI) to solve food problems has become prevalent in recent years as AI not only offers the possibility of optimizing and automating processes but also effectively reduces human error and labor costs [12,13,14,15]. An important branch of AI is Machine learning (ML), which aims to design and develop algorithms to give computers learning properties [16]. ML commonly uses simple or fixed features to achieve image classification and has the disadvantage of being dependent on manually designed feature extractors for classification results [17]. In addition, simple features are hardly representative of the class properties of an image. In contrast, the most attractive and efficient ML approach currently available is deep learning (DL) because DL has significant advantages in automatically learning data representations, e.g., automatic feature extraction from images [18]. DL algorithms have been successfully applied to various food domains, including food recognition, calorie estimation, nutrient or dietary assessment and quality inspection [19,20,21]. Moreover, two studies have reported the application of DL methods in detecting and counting oysters [22] or oyster larvae [23] since 2021, but to the best of our knowledge, no study has reported the use of DL method to predict oyster freshness as well as to investigate the interpretability of DL-based models for oyster applications.

According to the literature review, convolutional neural network (CNN) has become the most widely used DL algorithms for image-based identification, classification and detection tasks in the food field [24,25]. With its excellent ability to automatically learn features from images, CNN-based approaches have been integrated with existing seafood practices to accelerate the development of smart aquaculture [26,27]. In addition, in our previous studies, we have successfully employed several CNN-based methods to solve the engineering problems in experimental or food practices. For example, an AlexNet-based model was applied to identify and quantify three physical mechanisms of oil-in-water emulsions [18]; three CNN-based models (AlexNet, VGG-16 and VGG-19) were employed to quantify two morphological characteristics of spray-dried microcapsules [28]; and a DenseNet-101-based model was used to build a generic intelligent tomato classification system for practical applications [24]. Through these studies, we found that the AlexNet-based model has impressive advantages for applications in the food domain, including fast training and testing times, high accuracy and the potential to clearly generate feature visualization and strongest activation images, all of which helped us to better understand the mechanisms of how DL models work in the food domain.

Based on the above, this study proposes an intelligent method for predicting the freshness of Pacific oysters in a two-tier parallel combinatorial architecture. The method incorporates DL-based models and a polynomial fitting model to predict malondialdehyde, total sulfhydryl group and storage day for oysters in images. The contribution of this study is not only limited to proposing an automatic, efficient and accurate method for the non-destructive detection of oyster chemical indicators but also demonstrates the feasibility of applying the fusion of DL models and polynomial fitting model in the food production and processing, combined with the interpretability techniques of “black box” model, which contributes to the development of the food industry from the perspective of interdisciplinary applications of computer vision and artificial intelligence.

## 2. Materials and Methods

### 2.1. Sample Preparation and Storage

Commercially available Pacific Oysters (*Crassostrea gigas*) were farmed in Rongcheng (Weihai, China), with an average length, width and depth of 103.34 ± 12.34 mm, 53.33 ± 5.44 mm, 32.51 ± 4.62 mm, respectively. The average total wet weight of oysters and oyster meat were 99.91 ± 13.96 g and 21.06 ± 4.08 g, respectively. Fresh oysters were immediately shucked on the half shell and then stored at 4 °C for 0, 2, 4, 6, 8, 10, 12 and 14 days. Thus, a total of eight batches of oysters on the half shell were obtained, with each batch containing 35 oysters.

### 2.2. Biochemical Tests

The contents of malondialdehyde (MDA) and total sulfhydryl group (SH) of oyster meat at different storage days were determined to assess the freshness of oysters, i.e., to characterize the degree of lipid oxidation and protein structural integrity of oyster meat, respectively. Oyster meat with normal saline was first homogenized using Tissue-Tearor (BioSpec Products Inc., Bartlesville, OK, USA) at a ratio of weight (g): volume (mL) = 1:9, and the homogenate was centrifuged at 3000× *g* for 10 min at 4 °C to obtain supernatant. Then, the MDA and SH content in the supernatant of each sample were determined separately using commercial MDA and SH kits (purchased from Nanjing Jiancheng Bioengineering Institute, Jiangsu, China), according to the manufacturer’s instructions [29,30].

### 2.3. MDA-SH-Storage Days Polynomial Fitting Model Construction

Based on the experimental data obtained in Section 2.2, a polynomial fitting model of MDA (*x*)-SH (*y*)-Storage Days (*z*) was constructed using a MATLAB (version: R2021a) curve-fitting-tool to describe their relationship.

### 2.4. Oyster Meat Image Dataset Build-Up

#### 2.4.1. Image Acquisition

**Figure 1:**
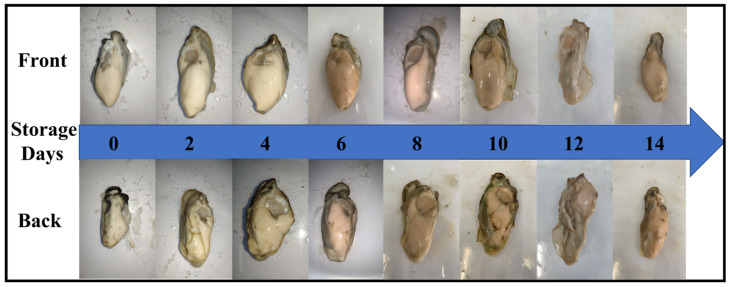
Example images of oyster meat at different storage days.

For the purpose of making the proposed method more user-friendly under routine laboratory conditions and easy to scale up, we used the cameras of ordinary mobile phones to capture images in natural light. The images of oyster meat at different storage days were randomly captured by iPhone XS Max, iPhone 7 Plus, Oppo R11 and Xiaomi Redmi Note 9 pro. Four images were taken for each oyster, two for the front and two for the back; 140 images were taken for each batch; and a total of 1120 images were obtained. Image properties are shown in Table 1, and the example images of oyster meat are shown in Figure 1. As can be seen in Figure 1, fresh oysters that have been stored for a short period of time are green, shiny and have fuller bodies whereas oysters that have been stored for a longer period of time are darker and drier.

As the oyster freshness was assessed by MDA and SH content of oyster meat at different storage days, this work involves two classification tasks based on oyster meat images. Task 1 is to predict the MDA value of oyster meat, and Task 2 is to predict the SH value of oyster meat, i.e., the oyster meat images need to be labeled with MDA and SH values, respectively. Specifically, taking the MDA values as an example, all the MDA values obtained in Section 2.2 were first sorted in ascending order, then divided into 4 levels according to the data balance principle and their median values were determined, and finally the 4 median values were used to label the images of the corresponding groups. Based on this, Task 1 was transformed into a 4-class classification task. Unlike the MDA values, all the SH values were first sorted in descending order and then the same steps were performed to transform Task 2 into a 4-class classification task as well.

Subsequently, for each task, a total of 1044 labeled images were randomly selected to construct training, validation and testing datasets for testing the performance of the proposed method; the detailed configuration of the datasets is presented in Table 2. In addition, the training and testing datasets were independent of each other in order to validate the stability and reliability of the proposed method objectively and effectively.

#### 2.4.2. Image Resizing

All images were resized to 227 × 227 pixels to meet the input image size requirements for the input layer of AlexNet-based classification model.

### 2.5. Deep Learning-Based Model Construction

AlexNet, proposed by Krizhevsky et al. [31], is a large CNN with 60 million parameters and 650,000 neurons. It is a series network with 25 layers arranged one after another, which includes, for example, an input layer, an output layer, five convolutional layers, three max-pooling layers and three fully connected layers [32]. Two classification models were built in this paper due to the fusion of MDA and SH content information, named AlexNet-MDA and AlexNet-SH. In addition, transfer learning was also employed in the training process of the proposed models. More details regarding AlexNet and transfer learning can be found in our previous studies [18,28].

### 2.6. Metrics for Performance Evaluation of Classification Models

In the DL field and statistical classification problems, a confusion matrix is used as a visualisation tool to represent the performance of a model on each category using a matrix of *n* rows and *n* columns. In addition, common metrices including accuracy, precision, recall, specificity and F1 score also can be calculated from the confusion matrix [33]. Therefore, the confusion matrix and these metrices were presented in this study to evaluate the overall performance of the proposed model. Formulas of the presented metrices were referenced from Fawcett’s paper [34,35].

### 2.7. Feature Visualization and Strongest Activations

To improve the interpretability of the DL-based “black box” model, the “*Deep Dream Image*” method and the “*Visualize Activations of a Convolutional Neural Network*” method were used to generate feature visualization images of the last fully connected layer and strongest activations images of the last convolutional layer of the two trained classification models, respectively. More details regarding this section can be found in our previous studies [18,28].

Taking feature visualization as an example, deep learning is a “black box” issue, and its interpretability has always been a hot topic of research, which is closely related to the level of user trust in the model [36]. As CNNs have the ability of automatically learning and extracting features from images in the training dataset and applying them to classification tasks, generating feature visualization images of the model during human interaction with the CNN model can give the model a certain level of interpretability, i.e., demonstrates the trained model’s understanding of different levels of oyster meat images to help people understand the working mechanism of the CNN and provide a better interactive experience.

## 3. Results and Discussion

### 3.1. Computer Configuration and Operating Parameters

A personal computer with Intel(R) Core i9-10900k CPU*1, 32 GB memory*1 and NVIDIA GeForce RTX 3090 GPU*1 was employed in this study. MATLAB R2021a version was used to develop and perform the two AlexNet-based models, and the models were trained by Adaptive Moment Estimation (ADAM). In addition, the hyperparameters were adopted: Initial learning rate was 0.00001; learn rate drop factor was 0.1; learn rate drop period was 10; minibatch size was set to 64; and max epoch was set to 15.

### 3.2. MDA-SH-Storage Day Polynomial Fitting Model

The mean values of MDA (*x*) and SH (*y*) contents of the eight batches and the corresponding storage days (*z*) were used to fit a model; the data is shown below.

*x (nmol/mgprot)* = [4.44, 4.84, 5.01, 5.99, 6.18, 6.74, 6.81, 7.24]

*y (μmol/gprot)* = [150.43, 134.09, 124.89, 112.16, 104.87, 103.25, 101.67, 98.17]

*z (day)* = [0, 2, 4, 6, 8, 10, 12, 14]

The polynomial fitting model is:

(1) $$z=-1.779x^{3}-0.8836x^{2}y+43.52x^{2}+0.9794xy-319.3x-2.968y+769.8$$

Coefficients with 95% confidence interval, Goodness of fit: R^2^ = 0.9946.

**Figure 2:**
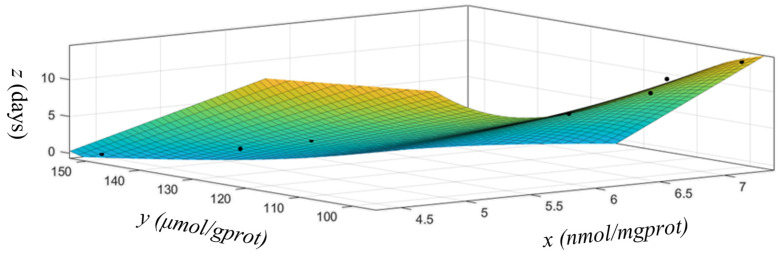
The MDA (*x*)-SH (*y*)-Storage days (*z*) polynomial fitting model.

The image of the polynomial fitting model is shown in Figure 2, and the median values for the four levels used to label the oyster meat images are shown in Table 3.

The biochemical reactions that occur in oyster meat during low temperature storage have been reported to increase with storage time and lead to a gradual deterioration in the quality of oyster meat [37]. Thus, an increase in MDA content indicates an increased level of lipid oxidation in oyster meat while a decrease in SH content was associated with chemical changes in the protein, which was probably caused by protein oxidation [38,39].

### 3.3. Performance of AlexNet-Based Classification Models

**Figure 3:**
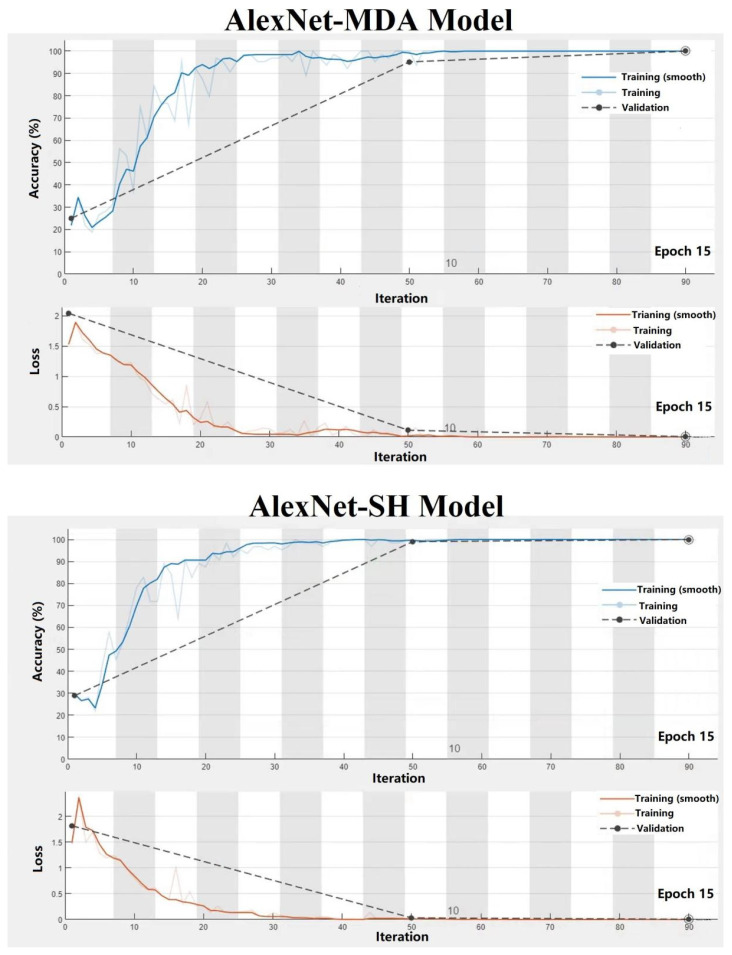
Training progress of AlexNet-MDA and AlexNet-SH models.

Training progress image is generally used to show the changes in classification accuracy and cross-entropy loss of the models during the training process. Figure 3 shows that the accuracy and cross-entropy loss of the two AlexNet-based models on the training set leveled off from fluctuations as the number of iteration and epoch increased and reached the highest and lowest values at 15th epoch, respectively. Based on this, the maximum epoch was set as 15.

**Figure 4:**
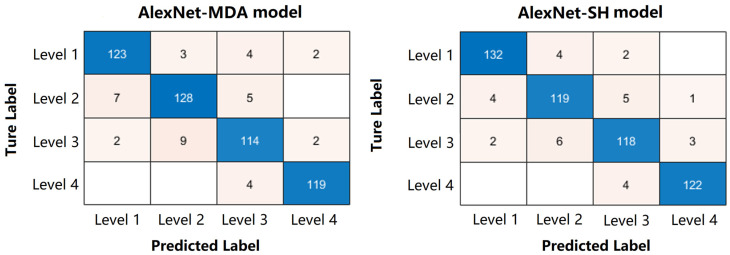
Confusion matrix for testing dataset of AlexNet-MDA and AlexNet-SH models.

The confusion matrix (Figure 4) and the common metrics (Table 4) were used to evaluate the performance of the two trained classification models in determining different levels of oysters. The confusion matrix of AlexNet-MDA model is taken as an example; the correct predictions for each category are located on the diagonal of the table. Specifically, the first column shows that a total of 132 images in the testing dataset were determined by the trained model to be the Level 1 category, where 123 images were actually classified accurately, and 7 and 2 were classified incorrectly and actually belong to Level 2 and Level 3, respectively. Thus, the precision for these 4 Levels was 93.18%, 91.43%, 89.76% and 96.75%, respectively. Meanwhile, as shown in the first row, a total of 132 images that belonged to the Level 1 category, 123 of which were correctly predicted, but the remaining 3, 4 and 2 images were incorrectly predicted by the trained model as Level 2, Level 3 and Level 4 categories, respectively. Therefore, the recall (sensitivity) for these 4 Levels was 93.18%, 91.43%, 89.76% and 96.75%, respectively.

Moreover, the overall accuracies of the AlexNet-MDA and AlexNet-SH classification models were 92.72% and 94.06%, respectively. The overall accuracies above 90% indicate that the two proposed AlexNet-based classification models are suitable for classifying oyster meat images. Furthermore, both models were developed and utilized on a personal computer with a training time of around 11 s and a testing time of around 0.3 s. The shorter training and testing times show that the proposed classification models are highly efficient, fast running, less expensive and have greater potential for practical applications.

### 3.4. Feature Visualization

**Figure 5:**
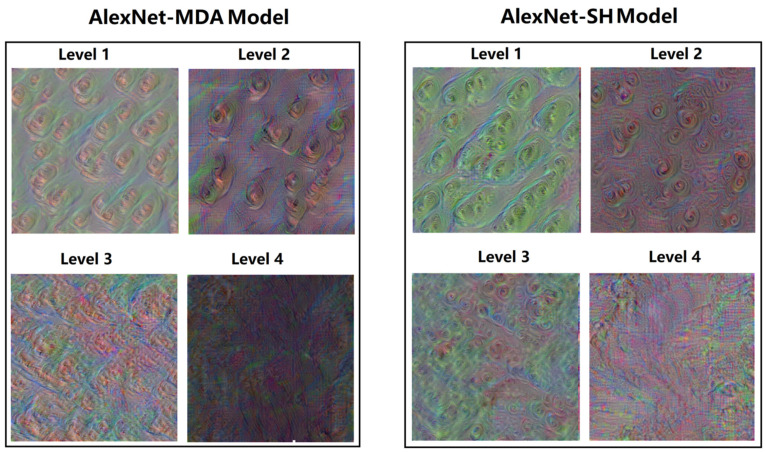
Feature visualization images of AlexNet-MDA and AlexNet-SH models.

Figure 5 is the feature visualization image of the last fully connected layer of the two trained classification models. This layer is near the end of the network and has deeper layers to learn and integrate the simple or low-level features learned by the previous layers into high-level features and use them in the classification task. The shallower layers generally learn simple features including lines, textures, edges, colors or shapes while the deeper layers generally learn more complex and abstract high-level features, including patterns or parts, which are sometimes difficult to describe and understand [28,40]. As shown in this figure, both trained classification models produce feature visualization images that are colorful but abstract, probably due to the diverse and irregular morphology of oyster meat and the large variation in individual morphology. Also, the Level 1 and Level 2 images have multiple similar irregular patterns while the Level 3 and Level 4 images are more abstract and blurred. In particular, the Level 4 feature visualization image consists of large blurred blocks of color with no discernible pattern. This may be due to changes in the surface morphology of the oyster meat as storage time increases, as it was found that oyster meat stored for longer periods of time had a cloudy appearance with a large amount of mucus on the surface and blurred tissue structure during image acquisition.

### 3.5. Strongest Activations

**Figure 6:**
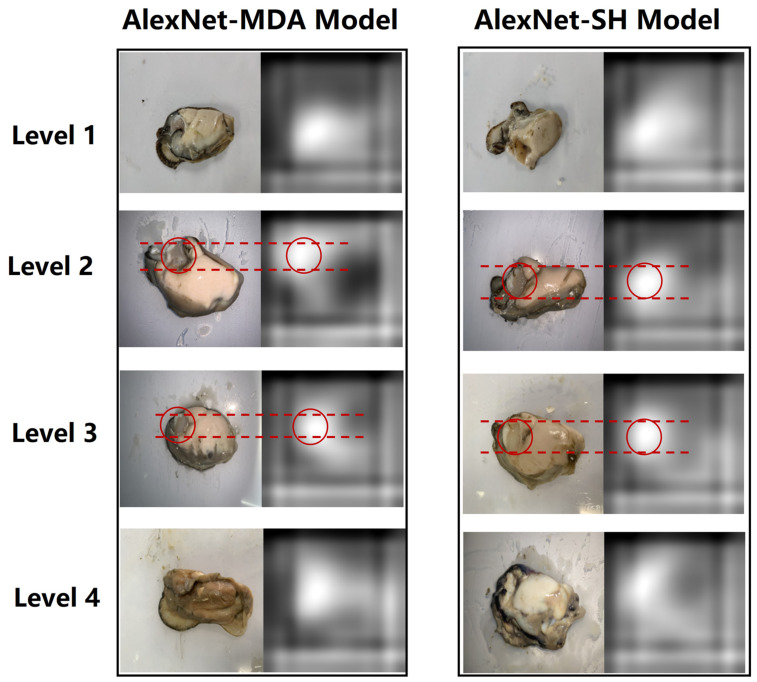
Strongest activations images of AlexNet-MDA and AlexNet-SH models.

One oyster meat image from each class in the testing set was randomly selected and fed into the trained model to generate the strongest activation image for the last convolutional layer of the model, as shown in Figure 6, with the image to the right of the oyster meat being its corresponding strongest activation image. The purpose of this part of the work was to show the basis on which the trained model identifies and classifies the different types of oyster meat images. The black pixels in the strongest activation image represent negative activation, and the white pixels represent positive activation. We focus on the strong white areas as they represent the features that the model relies on to recognize the different oyster images [24]. Interestingly, a comparison of the oyster images with their corresponding strongest activation maps shows that both models identified locations near the oyster’s adductor muscle, as shown by the red dashed line, meaning that the models may have classified the oysters by identifying around the oval adductor muscle. This phenomenon inspired us to further investigate the freshness changes of adductor muscle with increasing storage time, as this location may be a strong indicator of oyster freshness, or it may have an important role in causing changes in the freshness of the surrounding tissues.

### 3.6. Application of the Proposed Methods

**Figure 7:**
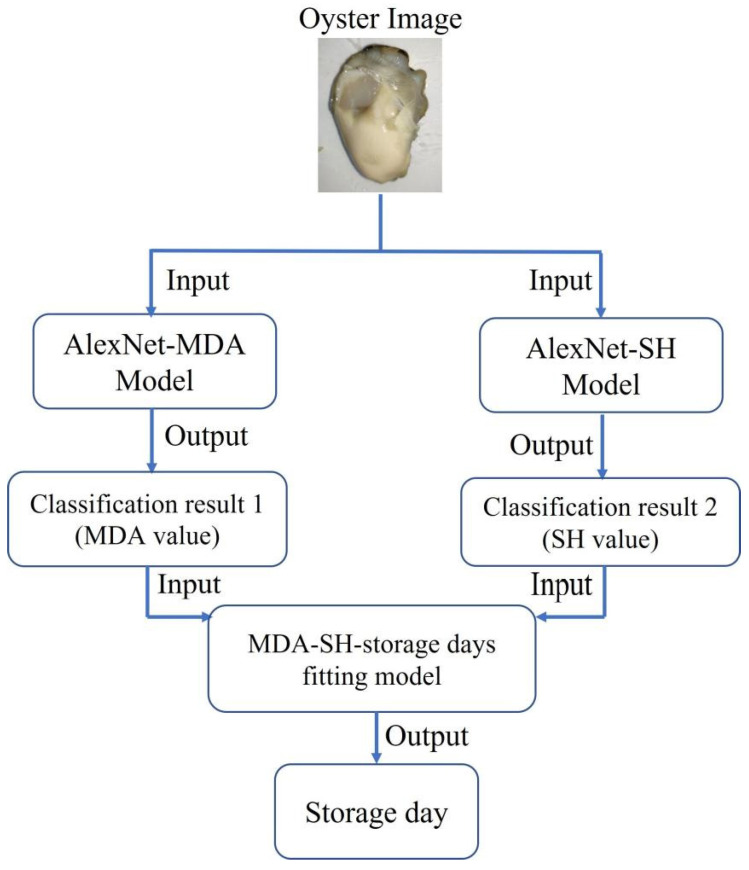
Flow chart of applying oyster freshness prediction model.

Based on the above, we designed a two-tier parallel combinatorial architecture for predicting oyster freshness, as shown in Figure 7. By randomly feeding oyster meat images into this architecture, the corresponding MDA content, SH content and storage day of the oyster in the image can be output within 0.03 ms on an ordinary computer, achieving a fast prediction of oyster freshness. As shown in Table 5, ten oyster images were randomly selected for input into the model and the corresponding predicted values were obtained. Although the predicted storage days were slightly different from the actual storage days, the results obtained were useful for oyster freshness prediction and demonstrated the feasibility of applying deep learning methods to oyster freshness prediction. Since deep learning is a data-driven approach, the performance and running speed of the proposed method will be further improved as the data size is scaled up and the computer hardware is upgraded. In addition, the method has no strict restrictions on the image capture environment, capture equipment and image clarity, which provides a new non-destructive method for monitoring and analyzing the quality of oysters during circulation, as well as new insights and ideas on the freshness changes of oysters during low temperature storage from the perspective of computer vision and artificial intelligence.

## 4. Conclusions

Since traditional oyster freshness detection methods are complicated to operate and difficult to quickly achieve non-destructive batch testing of oyster quality, this study proposed a new method for intelligent prediction of oyster freshness based on deep learning. In this study, (1) MDA and SH of oyster meat at different storage days were measured to assess freshness, and an “MDA-SH-storage days” polynomial fitting model was constructed. (2) Oyster meat images of different storage days were collected, labeled with MDA and SH information, and two AlexNet-based classification models were constructed to identify and classify oyster images of four different levels. (3) The outputs of the two classification models were used as inputs to the “MDA-SH-storage days” polynomial fitting model to form a two-tier parallel combinatorial architecture for oyster freshness prediction. (4) The understanding of the oyster meat images by classification models is shown in two visualisation ways to explore the interpretability of deep learning in food domain. Therefore, the results obtained above provide a theoretical and practical basis for the further applications of deep learning and its related work in the food field in the future.

## Figures and Tables

**Table 1:** Image properties of oyster meat captured by different mobile phones.

Camera	Pixels	Resolution (Horizontal/Vertical)	Bits Depth
iPhone XS Max	3024 × 4032	72 dpi	24
iPhone 7 Plus	3024 × 4032	72 dpi	24
Oppo R13	3456 × 4608	72 dpi	24
Redmi Note 9 pro	3000 × 4000	72 dpi	24

**Table 2:** The number of images used for training, validation and testing of AlexNet-MDA and AlexNet-SH models.

	Dataset	Level 1	Level 2	Level 3	Level 4
**Task 1 (MDA)**	Training	106	112	102	99
	Validation	26	28	25	24
	Testing	132	140	127	123
**Task 2 (SH)**	Training	111	104	104	101
	Validation	27	25	25	25
	Testing	138	129	129	126

**Table 3:** Median of the MDA and SH content values in different groups.

Group	Level 1	Level 2	Level 3	Level 4
MDA (nmol/mgprot)	3.30	5.15	6.25	10.20
SH (μmol/gprot)	163.98	119.19	106.42	66.37

**Table 4:** Performance evaluation metrics for AlexNet-MDA and AlexNet-SH models and their training and testing times.

Precision (%)			Recall (%)	Specificity (%)	F1 Score	Accuracy (%)	Training Time (s)	Testing Time (s)
**Task 1** AlexNet-MDA	Level 1	93.18	93.18	99.23	0.93	92.72	11.01	0.30
	Level 2	91.43	91.43	96.86	0.91			
	Level 3	89.76	89.76	96.71	0.90			
	Level 4	96.75	96.75	99.00	0.97			
**Task 2** AlexNet-SH	Level 1	95.65	95.65	98.44	0.96	94.06	11.14	0.32
	Level 2	92.25	92.25	97.46	0.92			
	Level 3	91.47	91.47	97.20	0.91			
	Level 4	96.83	96.83	98.99	0.97			

**Table 5:** MDA (*x*), SH (*y*) and storage day (*z*) output by oyster freshness prediction model.

Image	MDA (*x*)	SH (*y*)	Predicted Storage Day (*z*)	Actual Storage Day
1	5.15	119.19	4.77	4
2	6.25	106.42	8.10	8
3	5.15	163.98	−7.21	0
4	10.20	66.37	8.78	10
5	3.30	163.98	11.62	2
6	6.25	119.19	4.29	4
7	5.15	106.42	8.19	8
8	10.20	66.37	8.78	10
9	6.25	66.37	20.05	14
10	6.25	119.19	4.29	4

## Data Availability

The data presented in this study are available on request from the corresponding author or first author.
